# An evidence double standard for pharmacological versus non-pharmacological interventions: Lessons from the COVID-19 pandemic

**DOI:** 10.1016/j.conctc.2023.101108

**Published:** 2023-03-11

**Authors:** Tracy Beth Høeg, Vinay K. Prasad

**Affiliations:** Department of Epidemiology and Biostatistics, University of California-San Francisco, 550 16th St 2nd floor, San Francisco, CA, 94158, USA

**Keywords:** Medical evidence, Public health, COVID-19, Pharmacological interventions, Non-pharmacological interventions

Throughout the COVID-19 pandemic, a wide range of interventions have been deployed with the goal of slowing viral spread or lessening harmful impacts of the virus after exposure or infection. These included either individual or society-wide interventions, and non-pharmacological or pharmacological interventions.

For pharmacological interventions, such as pills or injectable compounds, bioplausibility was derived from biochemical or in-vitro studies. For behavioral or non-pharmacological interventions, bioplausibility might have relied upon physics, aerosol science or simply that the intervention seemed logical.

Interventions can be embraced to different degrees. While some are merely recommended, others may be required or mandated. In general, there is a discrepancy in supporting evidence: Pharmacological interventions typically require numerous randomized studies before (or at least ongoing with) any recommendation, whereas non-pharmacological interventions were often untested prior to widespread adoption. A review [1] completed in August of 2021 found only around 1% of registered clinical trials on COVID-19 globally were for non-pharmaceutical interventions. This might be justifiable if pharmaceutical or pharmacological interventions had greater side effects or downsides, but this is often not the case.

For example, downsides of school closures have included but are not limited to increased school dropout rates [2], decreased academic achievement [3] and decreased lifetime earnings [4]. Downsides of sports closures may include weight gain [5] and negative effects on emotional well-being [6]. Downsides of interventions such as carbon dioxide (CO2) monitors or sensors may include money diverted from other evidence-based public health interventions, technological dependency, where focus is on the device rather than on situational awareness [7], heightened sense of anxiety about disease or, conversely, a false sense of security for those who are high risk [8,9].

For pharmacological interventions, side effects are predominately biological and often detectable, particularly in a randomized fashion. Conversely, for non-pharmacological interventions, the scope and magnitude of downsides are more difficult to quantify and may involve social, emotional, educational and often more delayed or difficult-to-define biological effects.

Because most biologically plausible interventions, even those supported by some observational data, are eventually shown to be ineffective in real-world conditions [10], we should presume the net harms of non-pharmacological and pharmacological interventions alike outweigh the benefits until high-quality evidence can be shown to the contrary.

As we look back on the COVID-19 pandemic, it is our obligation to identify ways we can improve our public health response in the future. While pharmacological interventions were more readily accepted or rejected based on rapidly completed high-quality trials, many non-pharmacological interventions with known or obvious harms and weak evidence at best continued to be recommended for years if not to this day.

In this essay, we contrast the varying response to two interventions: the use of ivermectin vs. C02 monitors against COVID-19 as examples of this evidentiary double standard. We then discuss the broader implications of this double standard during the COVID-19 pandemic highlighting why gathering high quality data quickly, preferably through randomized trials, should be required to guide public health recommendations and avoid policies with net societal harms in the future.

## 1 Ivermectin

Ivermectin is an FDA approved anti-parasitic drug. It has biological plausibility against SARS-CoV-2 because of its in vitro effects against RNA virus replication, including very promising [1] in vitro effects against SARS-CoV-2. Ivermectin relies on a chemical mechanism of action, and has been tested repeatedly in randomized trials, which thus far have rarely found significant benefits and have predominantly been negative.

There have been few [[11], [12], [13]]^,^ double blind, placebo-controlled RCTs that have found clinical benefit of ivermectin alone vs placebo. The vast majority of trials have been negative. Most trials have been underpowered to detect reductions in hospitalization or death of less than 30–50%. For example, the TOGETHER trial [14] found 17%, 23% and 12% reductions in hospitalization, mechanical ventilation and death, respectively, but none was significant. One systematic review and meta-analysis [15] from June of 2022 failed to identify benefit against severe disease or recovery time, but found, based on low certainty, it may reduce mortality (log OR -0.67[95% CI, −1.20 to −0.13]). Another systematic review of 25 randomized studies failed to identify a benefit against mortality or requirement for mechanical ventilation [16]. The ACTIV-6 trial [17] identified no significant clinical benefits of ivermectin, though it was completed in a setting of very high degree of population immunity. None of the randomized studies identified major safety issues with Ivermectin. Cost of individual ivermectin treatment would typically be less than $100. The FDA [18], CDC [19], NIH [20] and other public health and medical organizations [21,22] have not only not recommended ivermectin, but warned against its use for COVID-19.

## 2 CO2 monitors

Numerous experts have advocated for the use of personal CO2 monitors without randomized or high-quality data supporting their use against COVID-19. For example, one Chair of Atmospheric Chemistry, has advocated for [23], carrying a “CO2 sensor to determine where to sit in airports …” and has also recommended “measur[ing] CO2 levels when classes are running w[ith] people present … levels need to be < 800 ppm.”

Additional infectious disease experts have recommended [24] using personal CO2 devices to reduce the risk of COVID-19, admitting to carrying their own device. One coronavirus researcher explained he has a phone CO2 device which alarms when levels rise above a certain point, a sign ventilation is not sufficient to decrease his chance of getting COVID-19, at which point he puts on a mask [24].

Beyond the advice of experts to the public, some public schools in California are now, for the purposes of maintaining safety related to the COVID-19 pandemic, required to use CO2 sensors in some classrooms [25]. 10.13039/100004956Minnesota Department of Health recommends their use in high-occupancy classrooms [26]. The CDC supports the use of portable CO2 monitors and recommends portable air cleaners for readings above 800 ppm [27].

CO2 monitors have been proposed as a way to minimize risk of contracting (or spreading) COVID-19. These monitors work through indirect mechanisms to estimate the amount of CO2 in the air. CO2 concentrations increase in an indoor space related to the number of people in a room, ventilation and, in certain circumstances such as during wildfires, surrounding environmental levels. The effectiveness of a CO2 monitor against COVID-19 relies on 1. Accurately measuring CO2 levels, 2. CO2 levels correlating with risk of infection, 3. A person's ability to react to a reading that is above their predetermined threshold of risk and 4. Assumes CO2 monitors give you information about an indoor environment you don't already know.

The monitors vary in terms of quality. To date, no randomized studies exist of rates of COVID-19 infection or transmission in different CO2 levels. One randomized trial [28] in a hospital found CO2 monitors placed in patient rooms with instructions to staff to keep CO2 levels below 800 parts per million did not result in significantly less time per day at elevated levels in the invention arm. The staff cited patient discomfort from cold with windows open as a major barrier to decreasing CO2 levels. This study demonstrates the important principle that even very plausible interventions will only work insofar as people can comply with them. Additional side effects of using CO2 monitors to determine or estimate COVID-19 infection risk include the need to change plans due to CO2 levels, increase in anxiety, false sense of security and potentially unnecessary expenses of an upgraded building ventilation system when an arbitrary CO2 threshold cannot be maintained. Higher quality personal CO2 monitors such as the ARANET4 recommended by one researcher cost around $250.

## 3 Recommendations and mandates of non-pharmacological interventions by public health authorities

Other non-pharmacological interventions have not only been recommended by public health authorities but, at times, mandated. In the United States, for example, the CDC ordered mask requirements for public transportation and required testing prior to international travel into the United States. The CDC also made recommendations for indoor masking and for certain conditions to be met for schools or businesses to reopen, which were then mandated on a local level.

## 4 Contrasting required levels of evidence for pharmacological vs non-pharmacological interventions

We conducted PubMed search on Feb 15th, 2023, to identify published randomized trials and a US National Library of Medicine search on ClinicalTrials.gov for ongoing/active randomized trials assessing the effects of ivermectin, hydroxychloroquine, convalescent plasma, CO2 monitors, masking, school, sports or business closures, and ventilation system changes against COVID-19. We excluded trials where the select interventions were studied only in combination with another intervention, where the outcomes did not include a measurement of efficacy against a COVID-19 endpoint or where the study was in preprint form only. We chose specifically not to use COVID-19 vaccines for comparison due that intervention's need for additional evidence to guide recommendations for different doses and in different populations.

**Fig. 1:**
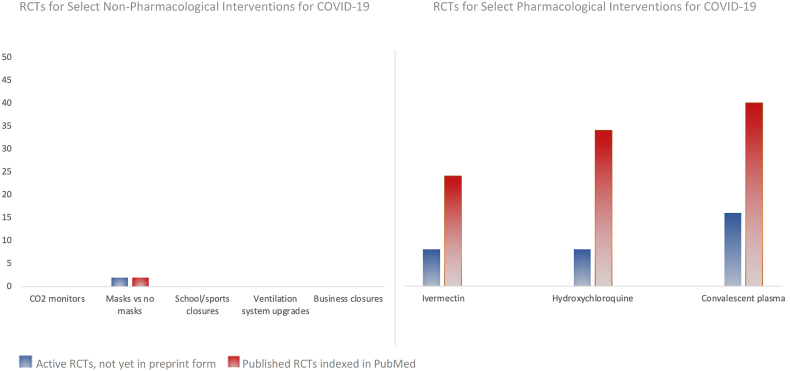
Ongoing and Published Randomized Controlled Trials for Select Non-Pharmacological vs Pharmacological Interventions for COVID-19.

In Table 1 and Fig. 1, we list pharmacological interventions, for which the medical community implicitly understands the importance of validation studies. It is not sufficient to have a mechanism of action; indeed it is merely a prerequisite. When it comes to non-pharmacological interventions, we do not extend the same scrutiny.

**Table 1:** Randomized investigation, mandates, risks and benefits of select non-pharmacological and pharmacological interventions against COVID-19.

Non-pharmacological interventions	Ongoing RCTs	Completed RCTs	Ever mandated?	Benefits demonstrated in randomized trials	Documented or theoretical risks
CO2 monitors	0	0	Yes, in some settings^a^	None^b^	Anxiety from being unable to decrease CO2 levels in certain situations, uncomfortable room temperatures due to need to open windows, cost, potentially needless upgrade to improved ventilation systems
Masks vs. no masks	2	2	Yes	1 RCT negative; 1 with modest surgical mask benefit only significant among ≥50 age group^c^	Interpersonal communication, word acquisition, comfort, anxiety about disease
School/sports closures	0	0	Yes	None^b^	Educational disruption, decreased lifetime earning potential, loss of safe environment outside of home, loss of fitness and socialization, disruption of physical, speech and other therapies
School/building ventilation system upgrades	0	0	Yes	None^b^	Cost to society/schools, increased energy demand compared with natural ventilation and potential movement away from outdoor air exposure
Business closures	0	0	Yes	None^b^	Loss of current and future earning potential, psychological stress of loss of business and productivity, unemployment costs and supply chain disruptions, delayed care, decreased life expectancy
**Pharmacological interventions**					
Ivermectin (alone)	8	24	No	Small minority of RCTs finding clinical benefit	Generally safe at recommended dosages
Hydroxychloroquine (alone)	8	34	No	No significant benefit found in existing RCTs	Generally safe at recommended dosages
Convalescent plasma	16	40	No	No significant benefit found in existing RCTs	Typically mild; similar to blood transfusion w/rare risk of infection or allergic reaction

a All public schools in California receiving CALShape grant money (https://forensicanalytical.com/blog/are-co2-sensors-now-required-in-all-california-classrooms/).

b Randomized evidence entirely lacking.

c This benefit has been called into question due to imbalances between treatment and control groups (https://trialsjournal.biomedcentral.com/articles/10.1186/s13063-022-06704-z).

Besides CO2 monitors, other examples include, but are not limited to, mask mandates, ventilation systems and school, sports and business closures. School closures, for example, were a massive societal intervention with numerous harms undertaken with a lack of evidence of effectiveness. Researchers from Norway famously called [29] for randomized studies of the intervention, which were not done, and their country's school closures were brief. For mask mandates in children, social and educational harms have been suspected and are now beginning to be documented [30].

It is not that randomized studies of non-pharmacological interventions cannot be done; they can, but are simply not expected or required by the medical and public health communities. We contend this is a dangerous and expensive double standard; we should require the same level of evidence for non-pharmacological interventions as pharmacological. High quality studies should be undertaken within weeks of a new pandemic to avoid unnecessary harms from ineffective interventions. Although non-pharmacological interventions have side effects that are more difficult to quantify and study, they should not be assumed to be of less importance and, in most cases, have wider societal consequences.

## Funding

None.

## Declaration of competing interest

The authors report no conflicts of interest. Both authors had access to the data and a role in writing this Commentary.
